# The Survival Rate from Splitting Clutch Design Method for Green Turtle’s Relocated Nest in Penang Island, Malaysia

**DOI:** 10.21315/tlsr2022.33.3.7

**Published:** 2022-09-30

**Authors:** Sarahaizad Mohd Salleh, Shahrul Anuar Mohd Sah, Ahmed Jalal Khan Chowdhury

**Affiliations:** 1 School of Biological Sciences, Universiti Sains Malaysia, 11800 USM Pulau Pinang, Malaysia; 2 Faculty of Agriculture, Universiti Islam Sultan Sharif Ali, Simpang 347 Jalan Pasar Gadong, BE1310 Brunei Darussalam

**Keywords:** Nesting Depth, Hatching Success, Survival Hatchlings, Splitting Clutch Design Method, Temperature, Kedalaman Sarang, Keberjayaan Penetasan, Anak Penyu Hidup, Kaedah Pengasingan Telur, Suhu

## Abstract

Ten nests were collected from Kerachut and Teluk Kampi, Penang Island between 2 August 2009 and 9 December 2009, and each one nest was split into three small clutch sizes for incubation at three nesting depths (45 cm, 55 cm and 65 cm), with a total of 30 modified nests for this experiment. Three important objectives were formulated; to observe on the survival hatchings among the three nesting depths, to study on the effects of sand temperature on incubation period among the three nesting depths, and to investigate the influence of sand temperature on hatchling’s morphology. Main result shows that the mean survival of the hatchlings was 25.40% at 45 cm nesting depth, followed by mean 17.60% at 55 cm nesting depth, and lastly, the mean was 21.50% at 65 cm nesting depth. Overall, there are 56.63% survival hatchlings, 10.97% dead hatchlings and 32.40% unhatched eggs were produced. The incubation period was also found to be significantly correlated with sand temperature, *p* > 0.001, and nesting depth, *p* < 0.001. The hatchling’s length and weight varies is sizes across the nesting depths, *p* < 0.001. However, the small difference in hatchling sizes per nesting depths are not strong enough to prove the significant correlation with sand temperature, *p* > 0.05. This article provides a basic knowledge from the splitting clutch design method. A sum of 50%–60% survivals hatchlings produced were incubating under small range of clutch sizes, 29 to 49 eggs. This article provides basic result on the survival hatchlings, eggs survivorship, incubation period, temperature, hatchling’s morphology and discussion on implication of this method on conservation in Malaysia.

## Abstract (fr)

Terdapat 10 sarang penyu yang dikutip di pantai Kerachut dan Teluk Kampi, di antara 2 Ogos 2009 hingga 9 Disember 2009, dan setiap satu sarang dibahagikan kepada tiga bahagian kecil yang sama rata untuk proses pengeraman pada tiga kedalaman sarang yang berbeza (45 cm, 55 cm dan 65 cm). Jumlah keseluruhan sarang yang diubahsuai untuk menjalankan eksperimen ini ialah sebanyak 30 sarang. Tiga objektif utama telah diketengahkan; dimana penelitian ke atas anak penyu menetas di tiga kedalaman sarang yang berbeza, untuk mengkaji kesan suhu sarang ke atas masa pengeraman di antara tiga kedalaman sarang yang berbeza, dan kajian pengaruh suhu sarang ke atas morfologi anak penyu yang menetas. Keputusan utama menunjukkan min anak penyu menetas adalah sebanyak 25.40% di kedalaman sarang 45 cm, diikuti min 17.60% di kedalaman sarang 55 cm, dan yang terakhir, min 21.50% di kedalaman sarang 65 cm. Secara keseluruhannya, terdapat 56.63% anak penyu menetas, 10.97% anak penyu mati dan 32.40% telur yang tidak menetas telah dihasilkan. Didapati bahawa tempoh penetasan mempunyai signifikasi korelasi ke atas suhu sarang, *p* > 0.001 dan kedalaman sarang, *p* > 0.001. Sementara itu, panjang anak penyu dan berat anak penyu didapati berbeza saiz mengikut kedalaman sarang, *p* < 0.001. Namun begitu, perbezaan saiz anak penyu yang tidak begitu ketara yang dihasilkan mengikut kedalaman sarang tidak memberi kesan ke atas signifikasi korelasi dengan suhu sarang, *p* > 0.05. Kajian ini memberikan maklumat asas keputusan daripada kaedah pengasingan telur. Jumlah keseluruhan menunjukkan 50%–60% anak penyu menetas berjaya dihasilkan, walaupun dieramkan dalam jumlah kumpulan telur yang kecil, iaitu sebanyak 29–49 telur. Artikel ini adalah penting untuk memberikan maklumat asas mengenai anak penyu menetas, survival telur, tempoh penetasan, suhu, morfologi anak penyu dan perbincangan implikasi kaedah ini kepada konservasi di Malaysia.

**Highlights**
- More than 50% survival hatchlings are produced from splitting clutch design method, even eggs were incubated under small clutch sizes that ranged between 29–49 eggs.
- Hatching success from splitting clutch design method at 45 cm prove to produce better hatching success than control nest in Penang Island.
- The mean of the hatchling’s length and weight are higher as the nesting depth increases, as temperature increased through increasing of nesting depths.

## INTRODUCTION

Eggs relocation programme is essential if *in-situ* method cannot be performed at some nesting beaches due to incontrollable eggs poaching problem, serious predator attack, location of nesting site that is exposed to serious flooding, and lack of staffs for patrolling the beach due to lack of budget (Mortimer *et al*. 1994). Eggs relocation programme started since 1995 in Penang Island, where between 3 and 65 nests were relocated from 1995 to 2009 to the hatchery of Kerachut Turtle Conservation Centre (Sarahaizad *et al*. 2012). The reasons are because the nesting beaches of Penang Island (especially Kerachut and Teluk Kampi) are exposed to natural predation (Fowler 1979; Hitchins *et al*. 2004; Chan 2010; Durmus *et al*. 2011; Peterson *et al*. 2013; Olgun *et al*. 2016), risky nest placement that located too close to the sea (Brown & Macdonald 1995; Madden *et al*. 2008), and due to nest exposure to high humidity from the sea water (López-Castro *et al*. 2004). In addition, early-stage hatchlings that emerged from nests are exposed to predator, which might excavate the nest and destroy the hatchlings that are yet to emerge from sand (Olgun *et al*. 2016). These refer to crabs (*Ocypode quadrata*), monitor lizard (*Varanus saluator*), smooth-coated otters (*Lutrogale perspicillata*) and Asian palm civet/common palm civet (*Paradoxurus hermaphrodites*) as a current observed land predators for sea turtle hatchlings and eggs in Penang Island. Consequently, almost more than 80% nests laid on the beach of Kerachut and Teluk Kampi were preferably incubated as relocated nests (Sarahaizad *et al*. 2012) due to above factors.

When the nests are relocated, there are various methods implemented to ensure the high-rate production of hatching success or hatching rate. The researchers do not advice on the eggs’ relocation programme (Mortimer 1999), however, suggest the nests to be exposed to a natural biodiversity condition (i.e., sand temperature, sand physical factors, topographic factors and sand chemical factors) for their natural hatching success production. Nevertheless, for some reason the eggs are advised to be relocated to a safer place due to unfavourable conditions as mentioned in the first paragraph. Therefore, various methods are implemented to ensure for a successful hatching success of relocated nests. The methods are:

1. Incubation according to nesting depth (Sarahaizad & Shahrul-Anuar 2014).
2. Incubation using Styrofoam box method (Mortimer *et al*. 1994; Abdul-Mutalib & Fadzly 2015).
3. Spliting clutch design method (Mortimer *et al*. 1994; Sarahaizad *et al*. 2017).

Previous study shows that eggs incubated at 55 cm nesting depth produce a better hatching success, than eggs incubated fewer than 65 cm or 75 cm nesting depths (Sarahaizad & Shahrul-Anuar 2014). This is because the temperature at 75 cm is higher, and able to break down the process and increase the eggs mortality (Sarahaizad & Shahrul-Anuar 2014), In addition, nests are also able to hatch when incubate inside the styrofoam box (Abdul-Mutalib & Fadzly 2015). An egg’s separation into two equal parts is believed to produce a high rate of hatching success (Mortimer *et al*. 1994; Mortimer 1999; Sarahaizad *et al*. 2017).

In this study, splitting clutch design method was performed, where the whole clutch size was divided into three parts, and incubate at three nesting depths (45 cm, 55 cm and 65 cm). The reason these depths conditions were chosen due to the natural range of nesting depth excavate by green turtle in most sand areas (Booth & Freeman 2006; Cheng *et al*. 2009). The rationale of the study is to calculate the survival hatchlings and eggs survivorship divisions from this splitting clutch design method. Are eggs able to survive when incubated under small clutch sizes of less than 50 eggs separately?

Three objectives are formulated for this study:

1. To observe on the percentage/number of survival hatching at three different nesting depth’s incubation.
2. To study on the effects of sand temperature on incubation period among the three nesting depths.
3. To investigate the influence of sand temperature on hatchling’s morphology.

Department of Fisheries Malaysia and Kerachut Turtle Conservation Centre initially performed this experiment. Therefore, the authors cooperated with the aforementioned departments to publish the accurate result, and with permission published the findings.

The splitting clutch design method has been previously conducted at Kerachut Turtle Conservation Centre, Penang Island by splitting the clutch size into two equal parts (Sarahaizad *et al*. 2018). This was the second study performed in Penang Island by using the same method by dividing one nest into three small clutch sizes. Hence, the publication of this outcome is important to reveal the result.

## MATERIALS AND METHODS

### Definition

*Control nests: In-situ* nests/natural nests.

*Modified nest*: Refer to nests that have been divided into three small clutch sizes.

*Clutch size*: Total eggs in one nest.

*Incubation period*: Total days it takes from the first day of incubation until first day of hatchling emergence.

*Splitting clutch design method*: Refers to the procedure of dividing whole clutch size into small parts of clutch sizes for incubation purpose.

*Survival hatchlings*: Refer to alive or aggressive hatchlings.

*Dead hatchlings*: Mortality of hatchlings.

*Unhatched eggs*: Refer to mortal or uncracked eggs.

### Study Sites

**Figure 1:**
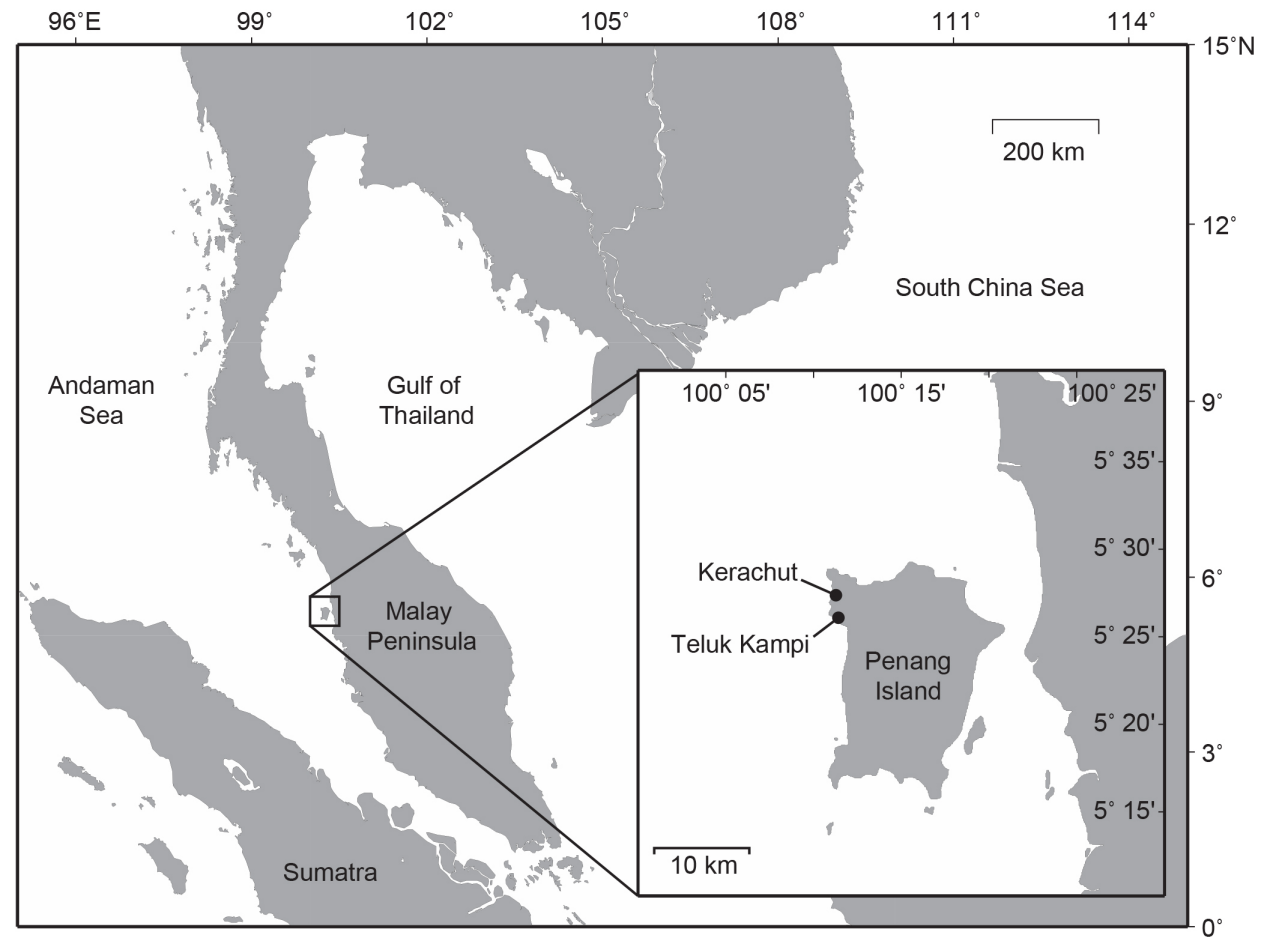
Nesting beaches of Kerachut and Teluk Kampi, located at Penang Island, Peninsular Malaysia. Ten nests were collected at both beaches before experiment was conducted inside the hatchery of Kerachut Turtle Conservation Centre. *Source*: Sarahaizad *et al*. (2018)

Turtle nests were collected at two nesting beaches, Kerachut (beach length = 558 m, longitude = 100.181, latitude = 5.451) and Teluk Kampi (beach length = 810 m, longitude = 100.179, latitude = 5.442) at Penang Island, Peninsular Malaysia (Fig. 1). Ten nests were collected from Kerachut and Teluk Kampi, between 2 August 2009 and 9 December 2009, relocated, and incubated in Kerachut Turtle Conservation Centre’s hatchery. The procedure of nocturnal survey (nest identifying on the beach) is similar to the procedure by Durmus *et al*. (2011) and Olgun *et al*. (2016), on the observation methods at night by four staffs hired by Kerachut Turtle Conservation Centre without disturbing the adult’s eggs depositing process. The eggs relocation procedure can be referred to the “Standard Procedure for Turtle Management Guidelines, Peninsular Malaysia” (Jabatan Perikanan Malaysia 2016) under relocated eggs section from article published by Sarahaizad and Shahrul-Anuar (2014). This includes the guideline on careful handling of eggs into the bucket, minimising the rotations of the eggs, sprinkling a little sand on the pile of eggs inside the bucket to match the temperature with the natural sand condition, and to minimise the shaking during the eggs transportation. Transportation was made as soon as possible to avoid delay of incubation.

### Control Nests

Ten (10) *in-situ* nests found at Kerachut were incubated naturally. Hatching success (%) for control nests was calculated according to the formula:


Hatching success (%)=(Total clutch size-number of unhatched eggs)/total clutch size×100 (Zare et al. 2012).

### Procedure of Splitting Clutch Design Method into the Three Small Clutch Sizes

Adult’s tag number, clutch size and date of incubation were recorded inside the record book prior commencing splitting clutch design method.

Clutch size was numerated and divided equally into three parts (for even number of eggs) or almost equal (for odd number of eggs), and incubate at three nesting depths (45 cm, 55 cm and 65 cm) (Table 1). Once divided into three parts, the modified nests were incubated at three nesting depths of 45 cm, 55 cm and 65 cm, and each modified nests were labelled with a bamboo plank (i.e., 1A, 1B, 1C; 2A, 2B, 2C; 3A, 3B, 3C). Information such as adult’s tag number, date of incubation, total eggs and expected date of hatching were written onto a bamboo plank before buried into each modified nest. The nests were protected with squared netlon mesh to avoid predator attack (Chan 2010). Sand temperature was monitored once a week for all modified nests using an electronic soil thermometer (±0.1 m) during daytime between 10:00 a.m.–12:00 p.m., and at the distance of 5 cm from sand’s surface (Sarahaizad & Shahrul-Anuar 2014). Three readings were deployed for each modified nests, and the mean were calculated to ensure the accuracy of sand temperature reading. This procedure was conducted for the next 10 nests examined (30 modified nests). Once the eggs began to hatch, the date of the first emergence of hatchlings was recorded. Incubation period was counted from the first day of eggs incubation until the first day of emergence hatchlings. Eggs survivorships division was calculated, and hatchling’s morphology was further measured.

### Eggs Survivorship

Total hatchlings found on sand’s surface were numerated and moved into the styrofoam box. Hatchlings that emerged by themselves were counted and were let to emerge by themselves for four days. On the fourth days of hatchling’s emergence, the nest was excavated to calculate the balance of alive dead hatchlings, and unhatched eggs inside the nest. Usually, nest excavation is conducted in the evening between 17:00 p.m. to 18:00 p.m. (the procedures of nest excavation to clean up the nests are referred to the standard procedure by Jabatan Perikanan Malaysia (2016).

Eggs survivorship were summarised by dividing into three categories; survival hatchlings, dead hatchlings and unhatched eggs. Survival hatchlings or alive hatchlings include a sum of hatchlings collected at sand’s surface and hatchlings found inside the nest. Dead hatchlings are referred to mortal hatchlings or deadly hatchlings found inside the nest. Based on data that received, embryonic eggs are not recorded in this experiment, therefore unhatched eggs or uncracked eggshells refer to a sum of embryonic eggs and infertile eggs. The definition and methods of calculating the survival hatchlings, dead hatchlings, and unhatched eggs were referred to the publication by Yalcin-Ozdilek and Yerli (2006), Durmus *et al*. (2011) and Olgun *et al*. (2016). The formulas for eggs survivorship (%):


$$\begin{array}{l} \text{Survival hatchlings}=\text{Number of alive hatchlings found on sand surface}+\text{Alive hatchlings found inside the nest }(\text{Chan }2013)\\ \text{Dead hatchlings}=\text{Total eggs in one nest}-(\text{Sum of survival hatchlings}+\text{Unhatched eggs})\\ \text{Unhatched eggs}=\text{Clutch size}-\text{Total number of cracked eggshells}\\ \text{Hatching success }(\%)=(\text{Total clutch size}-\text{number of unhatched eggs})/\text{total clutch size}\times 100\;(\text{Zare }et\;al.\;2012) \end{array}$$

### Hatchling’s Morphology

Twenty-five percent (25%) from the sum of survival hatchlings were separated, for a measurement of hatchling’s morphology (hatchling’s straight carapace length and hatchling’s weight). Hatchling straight carapace length was measured using an electronic Vernier slide caliper (±0.1mm) and measured from one end to end of hatchling’s carapace (Wood *et al*. 2014). Electronic weighting scale (±0.1 g) A3360-LT5001 Smith model was used to measure the hatchling’s weight. To ensure the accuracy, the weights of the hatchlings were measured, directly after collecting the hatchlings from the nest. Based on Hays *et al*. (2010) were avoided from putting them into the sea water before measurement, as hatchlings will turn into a “swimming frenzy” stage; thus, providing inaccuracy of reading.

### Statistical Analysis

Result was analysed using SPSS 17.0 version. In this study, a normality test, one-way ANOVA, Krukal-Wallis (K-W) test and Pearson’s correlation analysis were performed. As the data collected in this study was a large sample size, normality test, Kolmogorov-Smirnov (K-S) was performed. Normality test shows that the incubation period (K-S = 0.079, *df* = 30, *p* > 0.05) and sand temperature (K-S =0.108, *df* = 30, *p* > 0.05) data was normally distributed; therefore, one-way ANOVA was used to analyse the significant difference of these variables across the nesting depths. In contrast, normality test shows the data of survival hatchlings (K-S = 0.217, *df* = 30, *p* > 0.001), dead hatchlings (K-S = 0.169, *df* = 30, *p* < 0.05), unhatched eggs (K-S = 0.246, *df* = 30, *p* < 0.001), hatchling’s straight carapace length, (K-S = 0.509, *df* = 30, *p* < 0.001), and hatchling’s weight (K-S = 0.479, *df* = 30, *p* < 0.001) was not normally distributed. Therefore, Krukal-Wallis test (K-W) was performed to test these variables across the nesting depths. Pearson’s correlation analysis (large sample size) or Spearman’s correlation analysis (small sample size) was used to find a significant relationship between two continuous variables (Pallant 2002). In this case, Pearson’s correlation analysis was employed for the large sample size. Pearson’s correlation analysis was used to analyse correlation between (1) incubation period and nesting depths, (2) incubation period and sand temperature, (3) sand temperature and survival hatchlings, (4) hatchling’s straight carapace length and nesting depths, and (5) hatchling’s weight and nesting depths. Microsoft Excel was used to calculate the mean, standard deviation and graph design. An independent sample *t*-test was conducted to compare the means of hatching success between control and modified nests.

## RESULTS

### Clutch Size (Splitting Clutch Design Method)

Seven nests were collected at Kerachut and three nests were collected at Teluk Kampi, and the clutch size ranged from 90 to 147 eggs (mean ± SD = 113.90 eggs ± 17.63, *n =* 10). Four tagged turtles were identified; six nests were collected from these four tagged turtles, MY2574 (1 nests), MY2560 (2 nests), MY2572 (2 nests) and MY2573 (1 nests) (Table 1). In the meantime, another four nests were collected from the untagged turtles. A record of this untagged turtles (i.e., carapace measurement) was not identified; therefore, the estimation on the count of untagged turtles landing was unable to be identified. All 10 nests were divided into three small clutch sizes, and each modified nests had a small-clustered eggs that ranged between 29 and 49 eggs (mean ± SD = 37.97 ± 5.90, Table 2).

### Survival Hatchlings, Dead Hatchlings, Unhatched Eggs and Hatching Success

**Figure 2:**
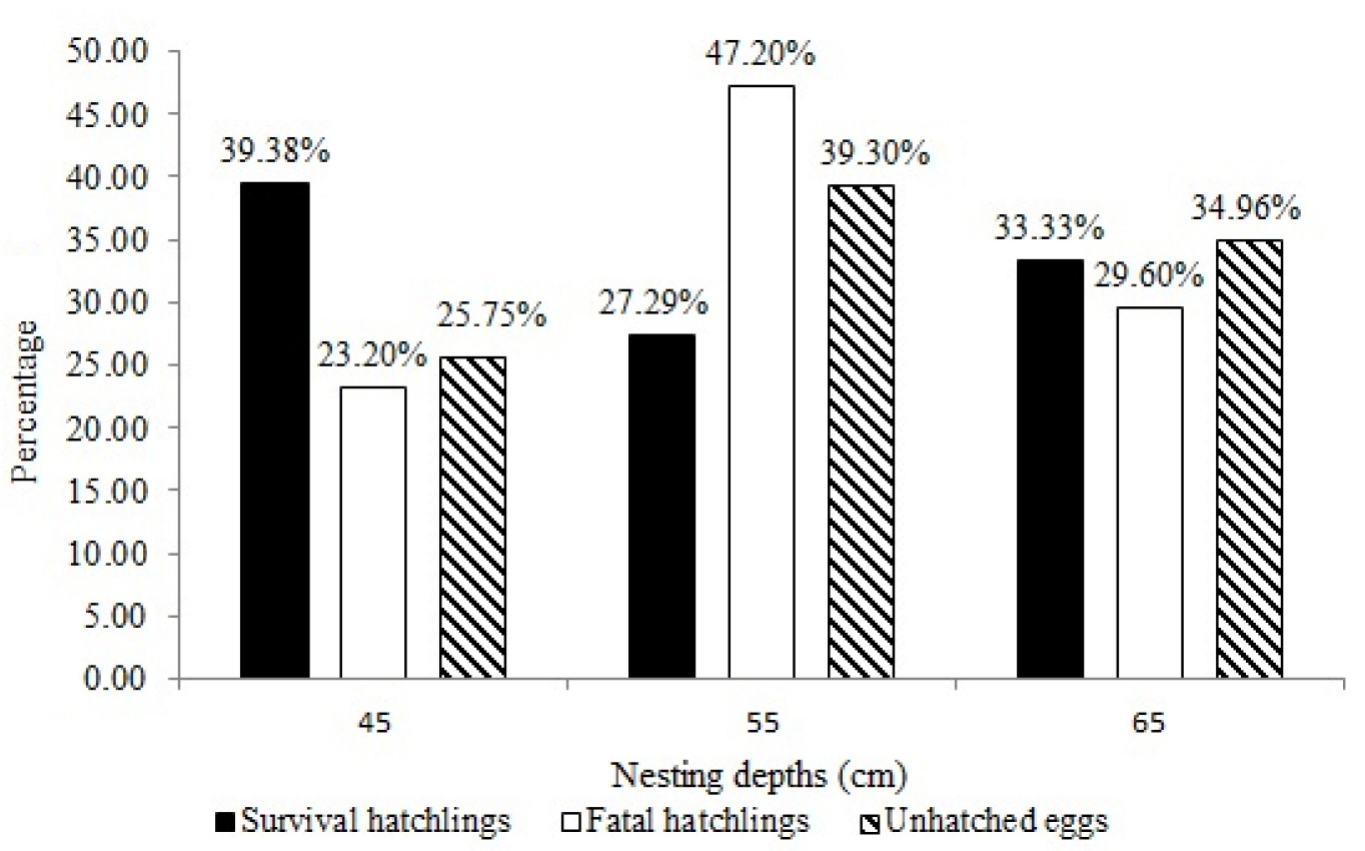
Survival hatchlings, dead hatchlings and unhatched eggs produced according to the nesting depths for 30 modified nests examined using splitting clutch design method.

At 45 cm nesting depths, a sum of 254 (39.38%) survival hatchlings were produced, and the mean survival hatchlings was mean ± SD = 25.40% ± 11.74, range = 0%–36%, as indicated in Fig. 2. This was followed by a sum of 176 (27.29%) survival hatchlings at 55 cm nesting depths, with mean ± SD = 17.60% ± 8.92, range = 1%–27%, and lastly a sum of 215 (33.33%) survival hatchlings were produced at 65 cm nesting depths, with mean ± SD = 21.50% ± 9.92, range = 1%–3%. There was no statistically significant difference of the percentage of survival hatchlings among the group of nesting depths, K-W = 5.406, *df* = 2, *p* > 0.05, *n* = 30, which mean the percentage of survival hatchlings produced were almost the same across 45 cm, 55 cm and 65 cm. The sand temperature does not affect the number of survival hatchlings produced as indicated by Pearson’s correlation coefficient (*r*) = −0.178, *n* = 30, *p* > 0.05.

For dead hatchlings record, 29 (23.20%) dead hatchlings were produced (mean ± SD = 2.9 ± 3.51, range = 0%–10%) at 45 cm nesting depth, followed by 59 hatchlings (47.2%) at 55 cm nesting depths (mean ± SD = 5.90 ± 5.30), range = 0%–17%), and 37 hatchlings (29.60%) at 65 cm nesting depths (mean ± SD = 3.70 ± 3.80, range = 0%–12%). While for unhatched eggs, 95 (25.75%) mortal eggs failed to hatch at 45 cm nesting depth, with a mean ± SD = 9.50 ± 12.81, range = 1%–41%. While at 55 cm nesting depth, 145 (39.30%) mortal eggs (mean ± SD = 14.50 ± 12.39, range = 3%–38%), and 129 (34.96%) mortal eggs at 65 cm nesting depths (mean ± SD = 12.90 ± 12.02, range = 2%–36%) were identified. The results on the detailed eggs survivorship for 30 nests are presented in Table 2. Percentage of dead hatchlings and unhatched eggs had almost similar distribution among the group of nesting depths, (dead hatchlings: K-W = 3.403, *df* = 2, *p* > 0.05, *n* = 30; unhatched eggs: K-W = 2.524, *df* = 2, *p* > 0.05, *n* = 30).

Overall, 645 (56.63%) survival hatchlings, 125 (10.97%) dead hatchlings and 369 (32.40%) unhatched eggs were produced from 1,139 eggs incubated. Overall hatching success is decreasing as increasing the nesting depth at 45 cm, 55 cm and 65 cm (Table 3).

### Hatching Success (%)

**Figure 3:**
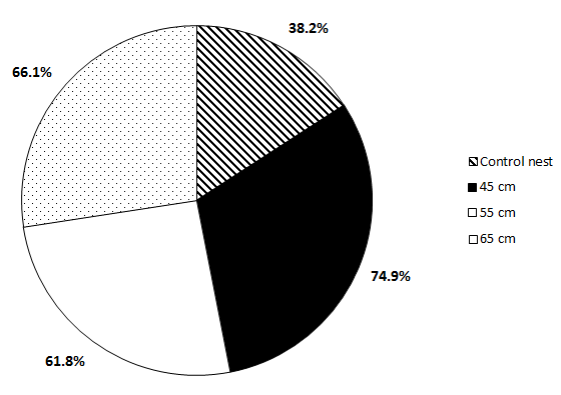
Mean hatching success comparison between modified nests and splitting clutch design method (at 45 cm, 55 cm and 65 cm).

We calculate the mean of hatching success between control nests (whole clutch size at various nesting depths) and split clutches at 45 cm, 55 cm and 65 cm. Control nest produces the mean of 38.2% of hatching success. While the highest mean hatching success produces at 45 cm or 74.9% (see Fig. 3), compared to at 55 cm and 65 cm. An independent sample *t*-test was conducted to compare the mean hatching success between control nests and modified nests incubated at 45 cm, and the was a significant difference between them, *t*(18) = −2.450, *p* < 0.05. But, not between modified nests incubated at 45 cm and 55 cm as the mean score of hatching success was almost uniformly distributed between them, *t*(18) = 0.542, *p* > 0.05.

### Incubation Period

Eggs were collected and incubated between 2 August 2009 and 9 December 2009, and hatched between 19 September and 5 February 2010 (Table 1). Incubation period ranged between 46 to 60 days (overall mean ± SD = 52.23 days ± 3.39); whereas at 45 cm, the mean incubation period was 55.2 days ± 2.6 (range = 52–60 days), followed by 52.2 days ± 2.14 (range = 49–56 days) at 55 cm, and lastly, 49.3 days ± 2.41 (range = 46–54 days) at 65 cm (Table 3). Distribution of incubation period was not the same among the three nesting depths as indicated by one-way ANOVA [*F* (2,27) = 13.718, *p* = < 0.001]. In addition, incubation period was significantly correlated with sand temperature, Pearson’s correlation coefficient (*r*) = −0.525, *n* = 30, *p* > 0.001, and nesting depth, Pearson’s correlation coefficient (*r*) = −0.710, *n* = 30, *p* < 0.001) which mean sand temperature and nesting depth influence the incubation period.

### Sand Temperature

At 45 cm nesting depth, the mean sand temperature was (mean ± SD = 28.23°C ± 0.71, range = 27.20–29.60°C), and at 55 cm nesting depth, the mean sand temperature was (mean ± SD 28.99°C ± 0.64, range = 28.10–30.40°C), followed by at 65 cm nesting depth, the mean sand temperature was (mean ± SD = 29.87°C ± 0.46, range = 29.00–30.60°C, Table 3). The result of the statistical analysis shows that the distribution of sand temperature was significantly different among the nesting depths, based on one-way ANOVA [*F* (2,27) = 16.072, *p* = < 0.001], which mean the sand temperature varies across the three nesting depths (increase depth increase the sand temperature). The mean sand temperature increased per nesting depths, and may influence the significant difference.

### Hatchling’s Morphology

A sum of 65 hatchlings were measured the hatchlings morphology at 45 cm nesting depths, and the results shows that the mean hatchling’s straight carapace length was (mean ± SD = 45.74 mm ± 0.04, range = 45.31 mm–45.96 mm), with the mean hatchlings’ weight at mean ± SD = 20.11g ± 0.11, range = 19.82 g–20.40 g. Furthermore, the measurement was taken at 55 nesting depth (a sum of 46 survival hatchlings were measured), and mean hatchling’s straight carapace length was (mean ± SD = 46.39 mm ± 0.11, range = 45.91 mm–46.68 mm), with the mean hatchlings’ weight was (mean ± SD = 20.63 ± 0.22 g, range = 19.98 g–21.07 g). Lastly, 55 hatchlings were measured at 65 nesting depths, and the mean hatchling’s straight carapace length was (mean ± SD = 46.76 mm ± 0.10, range = 46.41 mm–46.98 mm), with the mean of hatchling’s weight was (mean ± SD = 20.81 g ± 0.29, range = 20.17 g–21.35 g). The result is presented in Table 4. There was a statistically significant difference of hatchling’s straight carapace length produced among the group of nesting depths, K-W = 25.847, *df* = 2, *p* < 0.001, *n* = 30, and so the hatchling’s weight, K-W = 17.223, *df* = 2, *p* < 0.001, *n* = 30. This mean, the morphology sizes produced differed among the nesting depths. However, sand temperature did not correlate with hatchling’s straight carapace length, Pearson’s correlation coefficient (*r*) = 0.190, *n* = 30, *p* > 0.05, and with hatchling’s weight, Pearson’s correlation coefficient (*r*) = 0.220, *n* = 30, *p* > 0.05. Many factors affect the differences in the sizes of hatchling’s morphology produced (temperature, moisture content, sand particle size, grains size, sand’s chemical content factors), that include sand temperature. Table 4 indicate that small differences in hatchling’s morphology may not be strong enough to prove the correlation with sand temperature, as mean temperature also shows a different value among nesting depths.

## DISCUSSION

Even though the clutch size was split into small clutch sizes (< 50 eggs), the mean and range incubation period from this method was almost similar with the whole clutch size results at mean = 53 days in Turkey (green turtle, Olgun *et al*. 2016) and mean = 49.9 days in Cyprus (green turtle, Glen *et al*. 2005). This means that reducing the clutches are not really influencing the lessen days of times taken to reduce the incubation period.

The duration of incubation period was also influenced by ambiance sand temperature (Mortimer 1999; Hays *et al*. 2002; Chu *et al*. 2008; Maulany *et al*. 2012; Olgun *et al*. 2016), where higher temperature able to increase the metabolic rate and therefore lessen the duration taken for eggs to hatch (Booth 1998; Glen *et al*. 2005; Booth & Evans 2011). High temperature provides heat and important for growing the cell tissue, and expedite the metabolic process, and therefore, less time taken for eggs to hatch. Similar results were also found in the studies by Van De Merwe *et al*. (2006) and Wood *et al*. (2014). In addition, the surrounding temperature is suggested to increase due to natural pressure from the upper sand, air ventilation, air space, (Rusli *et al*. 2016), and complex interactions between chemical and physical (Van De Merwe *et al*., 2006), with deeper sand’s depth. Therefore, this explanation might be relevant to the result found, as nesting temperature varies across the nesting depth. The global trend of rising atmospheric temperatures poses many risks to the developing sea turtle embryo. Not only could continue atmospheric warming push incubation temperatures outside their viable developmental range, but clutch sex ratios and hatchling fitness may be negatively impacted. Therefore, the effectiveness of natural and artificial shade on reducing green turtle incubation temperature are worthy of future investigation (Reboul *et al*. 2021).

As clutch size had been split into small clutch sizes, the survival hatchlings were reduced and the number of survival hatchlings ranged between 0–36 hatchlings, with the overall mean of 56.63% survival hatchlings. In comparison to the hatching success produced without the splitting clutch design method, there are mean = 84.30% in Iran (mean clutch size = 124.00 eggs, hawksbill turtle, Zare *et al*. 2012) and mean = 55.51% in Turkey (mean clutch size = 72.66 eggs, green turtle, Olgun *et al*. 2016) A few researchers encourage performing splitting clutch design methods due to ability to provide high rate hatching success (Mortimer *et al*. 1994; Mortimer 1999), but in some point, other further discussion is needed to investigate the findings. For example, even though the percentage of survival hatchlings produced is normal, and does not show a sign of critical decrease, current relevant studies informed that reducing the clutch size will impact the increase risk of predator attack and lower down the energy reserved of newly hatchlings due to small group of survival hatchlings produced (Rusli *et al*. 2016).

Hatchlings prefer to emerge in groups (Carr & Hirth 1961). Naturally, the synchrony of turtle emergence hatchlings crawling from within a nest, is typically believed to decrease predation (Tucker *et al*. 2008) and is often used as a common example of the anti-predator role of grouping (Spencer & Janzen 2011) to prevent from predator attack. In this study, group of survival hatchlings had been reduced from splitting clutch design method (< 50 eggs), and small group emergence may affect the survival of small hatchlings.

The nest depths are the critical factor for the success of the *ex-situ* conservation method (Martins *et al*. 2007). Besides predation, nest depth is also an essential factor influencing the hatching and emergence success of green turtle, and there is a correlation between hatching success with the nest depth throughout the study by Najwa-Sawawi *et al*. (in press). However, the result is contradicted in this study as we found survival hatchlings are same across the depths. This study involved shorted nest depth between 45 cm–65 cm of relocated nests and the contradiction is because the study in Chagar Hutang Turtle Sanctuary involving natural observation for turtles digging a nests

The latest study reveals that splitting clutch design method also had a net negative issue since the reduced number of eggs in a clutch during incubation may eventually result in the production of hatchlings with reduced energy reserves when they enter the oceans (Rusli *et al*. 2016). This may affect their survival. Even good rate of hatching success was produced from splitting clutch design method, the future energy reserve is also important for survival during emergence, swimming in the sea, and protecting from ocean’s predator. The latest result shows that an increase in group size from 10 to 60 hatchlings are able to impact a ~50% decline in both the time taken to escape the nest, and resulting in reduced energy expenditure during nest escape (Rusli *et al*. 2016).

A significant difference of hatchling’s length and weight among the nesting depths are identified, which mean the morphology varies among the depths. The mean of the hatchling’s length and weight are higher as the nesting depth increases, as temperature increased through increasing of nesting depths (Table 3). Nest temperature influenced body size of hatchlings (Booth & Evans 2011). The elaboration is when eggs are exposed to high temperatures, the body tissue and complex cells inside the eggs will generate more actively during the embryonic development stage (Booth 2006; Booth & Evans 2011), therefore will elongate the sizes and weight of hatchlings.

Splitting clutch design method are good as between 50%–60% survival hatchling was produced, and from 30 modified nests, no abnormal hatchlings were produced from this method, as indicated in the previous studies (Sarahaizad *et al*. 2017). This percentage might be influenced by the surroundings sand temperature, which is considered as an optimal temperature recommended by Bustard and Greenham (1968), with a good rate of hatching success. However, this method may have implication in slowing down the hatchling’s energy reserved for swimming, locomotors performance, and reduced digging ability. These factors are able to influence the life survival and aggressiveness towards predator attack. Reducing the clutch size may affect the decrease in energy group of digging hatchlings. Therefore, by increasing the clutch size, the hatchlings may have longer survival in protecting themselves from predator attack.

Overall, we could see that control nests are producing less percentage of hatching success than modified nests. The highest mean hatching success was produced at 45 cm, in comparison to 55 and 65 cm, and the lowest hatching success is produced from the control nest. In the situation at Penang Island where the location is currently unsuitable for *in-situ* programme (far from the conservation centre, lack of staff, etc.), we suggest splitting clutch design method at 45 cm is applicable to reduce eggs mortality. In addition, it’s proven nests at a depth of 75 cm had significantly lower daily temperature ranges than nests at a depth of 50 cm (Van De Merwe *et al*., 2006), which could contribute to low metabolic rate and impact the hatch rate.

## CONCLUSION

This article provides a basic knowledge about the result from the splitting clutch design method into three parts. It shows that more that 50% survival hatchlings are produced, even eggs were incubated under small clutch sizes that ranged between 29–49 eggs (mean = 38 eggs). In addition, hatching success from splitting clutch design method at 45 cm prove to produce better hatching success than control nest in Penang Island. We suggest this method could be implemented at Kerachut Turtle Conservation Centre as the last choice to increase the hatching success if *in-situ* conservation is impossible to implement. Other successful research using splitting clutch design method were by Mortimer *et al*. (1994) and Mortimer (1999).

## Figures and Tables

**Table 1:** Clutch size, division of clutch sizes, nesting depth, turtle tagging number, date of incubation, date of hatching and incubation period.

No.	Nest no.	Clutch size	Location	Division of clutch sizes	Nesting depth (cm)	Turtle tagging no.	Date of incubation	Date of hatching	Incubation period (days)
1	1A	103	Kerachut	34	45	MY2574	2-Aug-09	29-Sep-09	58
	1B			34	55			23-Sep-09	53
	1C			35	65			19-Sep-09	48
2	2A	100	Kerachut	33	45	MY2560	7-Aug-09	31-Sep-09	55
	2B			33	55			28-Sep-09	52
	2C			34	65			26-Sep-09	50
3	3A	119	Teluk Kampi	39	45	Untagged	31-Aug-09	24-Oct-09	54
	3B			40	55			21-Oct-09	51
	3C			40	65			21-Oct-09	51
4	4A	147	Kerachut	49	45	MY2572	10-Sep-09	1-Nov-09	52
	4B			49	55			29-Oct-09	49
	4C			49	65			26-Oct-09	46
5	5A	124	Kerachut	41	45	Untagged	23-Sep-09	16-Nov-09	54
	5B			41	55			15-Nov-09	53
	5C			42	65			10-Nov-09	48
6	6A	106	Kerachut	34	45	MY2560	2-Oct-09	1-Dec-09	60
	6B			36	55			27-Nov-09	56
	6C			36	65			25-Nov-09	54
7	7A	98	Teluk Kampi	33	45	Untagged	12-Oct-09	4-Dec-09	53
	7B			33	55			1-Dec-09	50
	7C			32	65			28-Dec-09	47
8	8A	90	Teluk Kampi	31	45	Untagged	22-Oct-09	17-Dec-09	56
	8B			30	55			16-Dec-09	55
	8C			29	65			13-Dec-09	52
9	9A	112	Kerachut	37	45	MY2573	2-Nov-09	24-Dec-09	52
	9B			37	55			22-Dec-09	50
	9C			38	65			19-Dec-09	47
10	10A	140	Kerachut	47	45	MY2572	9-Dec-09	5-Feb-10	58
	10B			47	55			31-Jan-10	53
	10C			46	65			28-Jan-10	50

**Table 2:** Mean sand temperature (°C) and eggs survivorship per-nesting depths for 30 modified nests examined.

Modified nests no.	Artificial nesting depth (cm)	Mean sand temperature (°C)	Eggs survivorship						Division of clutch sizes

			Survival hatchings	%	Dead hatchings	%	Unhatched eggs	%	
1A	45	27.6	24	70.59	2	5.88	8	23.53	34
1B	55	28.4	24	70.59	0	0.00	10	29.41	34
1C	65	29.7	24	64.86	1	2.86	10	28.57	35
2A	45	28.2	30	90.91	0	0.00	3	9.09	33
2B	55	29.6	21	63.64	6	18.18	6	18.18	33
2C	65	30.3	25	73.53	2	5.88	7	20.59	34
3A	45	27.8	6	15.38	6	15.38	27	69.23	39
3B	55	28.3	1	2.50	4	10.00	35	87.50	40
3C	65	29.8	1	2.50	3	7.50	36	90.00	40
4A	45	29.6	35	71.43	8	16.33	6	12.24	49
4B	55	30.1	11	22.45	14	28.57	24	48.98	49
4C	65	30.5	5	10.20	12	24.49	32	65.31	49
5A	45	28.5	0	0.00	0	0.00	41	100.00	41
5B	55	29.6	3	7.32	0	0.00	38	92.68	41
5C	65	30.2	19	45.24	0	0.00	23	54.76	42
6A	45	28.1	32	94.12	1	2.94	1	2.94	34
6B	55	28.7	26	72.22	7	19.44	3	8.33	36
6C	65	29.3	30	83.33	3	8.33	3	8.33	36
7A	45	27.6	30	90.91	0	0.00	3	9.09	33
7B	55	28.2	24	72.73	3	9.09	6	18.18	33
7C	65	29.1	26	81.25	1	3.13	5	15.63	32
8A	45	29.2	28	90.32	1	3.23	2	6.45	31
8B	55	29.8	18	60.00	3	10.00	9	30.00	30
8C	65	30.4	25	86.21	2	6.90	2	6.90	29
9A	45	27.5	33	89.19	1	2.70	3	8.11	37
9B	55	28.6	21	56.76	5	13.51	11	29.73	37
9C	65	29.9	27	71.05	3	7.89	8	21.05	38
10A	45	29.0	36	76.60	10	21.28	1	2.13	47
10B	55	29.2	27	57.45	17	36.17	3	6.38	47
10C	65	30.4	33	71.74	10	21.74	3	6.52	46
Overall mean:		29.11	21.50		4.17		12.30		37.97
Sum:			645		125		369		1139

**Table 3:** Temperature (mean ± SD) deployed and incubation period (mean ± SD) calculated per nesting depths.

Nest depths (cm)	Temperature (°C)		Incubation period (days)		Overall hatching success (%)

	Mean	Range	Mean	Range	
45	28.23 ± 0.71	27.20–29.60	55.20 ± 2.60	52–60	74.87
55	28.99 ± 0.64	28.10–30.40	52.20 ± 2.14	49–56	61.84
65	29.87 ± 0.46	29.00–30.60	49.30 ± 2.41	46–54	66.10

**Table 4:** Hatchling’s straight carapace length (mean ± SD) and hatchling’s weight (mean ± SD) measured from 25% of survival hatchlings.

Nest depths (cm)	Total sample of survival hatchlings, 25% (*n*)	HSCL (mm)		Hatchling weight (g)	

		Mean	Range	Mean	Range
45	64	45.74	45.31–45.96	20.11	19.82–20.40
55	46	46.39	45.91–46.68	20.63	19.98–21.07
65	55	46.76	46.41–46.98	20.81	20.17–21.35
